# Up-regulation of bone morphogenetic protein and its signaling molecules following castration of bulls and their association with intramuscular fat content in Korean cattle

**DOI:** 10.1038/s41598-019-56439-2

**Published:** 2019-12-24

**Authors:** Da Jin Sol Jung, Myunggi Baik

**Affiliations:** 1 Department of Agricultural Biotechnology and Research Institute of Agriculture and Life Science, College of Agriculture and Life Sciences, Seoul National University, Gwanak-gu, Seoul 08826 Republic of Korea; 2 Institutes of Green Bio Science Technology, Seoul National University, Pyeongchang-gun, Gangwon 25354 Republic of Korea

**Keywords:** Reverse transcription polymerase chain reaction, Animal physiology

## Abstract

We evaluated whether castration affects bone morphogenetic protein 2 (BMP2) level and the expression of its signaling molecules in Korean cattle bulls. We also checked whether castration affects the expression of muscle fiber type and oxidative and glycolytic enzyme genes. Enzyme-linked immunosorbent assays revealed that steers had higher plasma BMP2 and leptin concentrations than bulls. Quantitative real-time PCR showed that steers had higher mRNA levels of the *lysyl oxidase* gene, a downstream target of the BMP signaling pathway, in the *longissimus thoracis* (LT) muscle. Steers had higher adipogenic *peroxisome proliferator-activated receptor gamma* and lipogenic *fatty acid binding protein 4* mRNA levels in the LT than bulls. Steers had lower mRNA levels for several muscle fiber type 1 genes and fiber type 2A myosin heavy chain 2 gene than bulls. Steers had higher mRNA levels of the glycolytic enzyme *phosphoglycerate kinase 1* gene than bulls. Transcript levels of oxidative enzyme genes did not differ between bulls and steers. Regression analysis revealed a positive association between plasma BMP2 levels and intramuscular fat (IMF) content in the steer group. These findings suggest that upregulation of the BMP signaling pathway in response to castration induces increased adipogenic gene expression, contributing to the increased IMF deposition observed in castrated animals.

## Introduction

Marbling refers to the dispersion or scattering of fat inside lean meat, and the degree of marbling is the major determinant of the quality grade of Korean cattle beef^1^. Castration significantly increases the marbling score (MS) and intramuscular fat (IMF) accumulation, improving beef quality in Korean cattle^1,2^. Previously, we showed that the activation of adipogenesis and lipogenesis is involved in increased IMF deposition post-castration^2–4^. Both hyperplasia (adipocyte number) and hypertrophy (adipocyte size) contribute to IMF deposition^5,6^. Adipocyte hyperplasia may result from new preadipocyte recruitment and commitment by mesenchymal stem cells (MSCs) from the vascular stroma of adipose tissue^7^, adipocyte proliferation via mitotic clonal expansion of the committed preadipocytes during differentiation^8^, and new adipocyte differentiation from preadipocytes^9^.

The bone morphogenetic protein (BMP) signaling pathway is thought to be required for the differentiation of MSCs into the adipocyte lineage based on cell culture studies^10–12^. *Lysyl oxidase* (*LOX*) is a downstream target gene of the BMP signaling pathway, and is implicated as an early marker of adipogenic commitment in cell culture^10^. Little is known about the involvement of BMP signaling molecules in marbling formation in bovine species. It is known that leptin can induce adipocyte differentiation in preadipocytes^13,14^. Limited information is available regarding whether castration affects plasma leptin levels.

Castration reduces circulating testosterone^2^ and affects muscle fiber types in cattle^15^. Thus, castration may affect the expression of genes involved in myofiber type composition and muscle metabolism through this endocrine change.

In this study, we aimed to understand changes in the expression of BMP2-adipogenesis signaling molecules caused by castration in cattle. We also evaluated whether castration of bulls affects the expression levels of muscle fiber type and metabolism-related genes.

## Results and Discussion

### Carcass characteristics of Korean cattle bulls and steers

The carcass weights of bulls and steers averaged 445 ± 9.37 and 434.3 ± 9.47 kg, respectively (Table 1). Steers had greater (p < 0.001) back fat thickness and greater (p < 0.001) MS and quality grade than bulls. Steer LT had a 4.0-fold greater (p < 0.001) IMF content (14.5 ± 5.48%) than bull LT (4.18 ± 1.69%). Bulls and steers were slaughtered at a similar weight, so steers were about 6 months older at slaughter. Age affects marbling development;^1^ therefore, in addition to castration itself, age differences may affect differences in MS and IMF content between bulls and steers.

**Table 1:** Carcass characteristics of the Korean cattle bulls and steers.

Variables	Bulls (n = 10)	Steers (n = 10)	P-value
Carcass weight, kg	445 ± 9.37	434 ± 9.47	0.42
Back fat thickness, mm	4.7 ± 0.63	11.7 ± 0.80	<0.001
Rib eye area, cm	86.4 ± 2.97	95.9 ± 6.89	0.23
Yield index^1^	69.0 ± 0.36	66.6 ± 0.97	0.04
Yield grade^2^	30.0 ± 0.00	22.0 ± 2.00	0.003
Marbling score^3^	1.1 ± 0.10	7.1 ± 0.35	<0.001
Quality grade^4^	11.0 ± 1.00	44.0 ± 1.63	<0.001
IMF content, %	4.18 ± 1.69	14.5 ± 5.48	<0.001

Values are mean ± standard deviation.

^1^Yield index = 68.184–0.625 × back fat thickness + 0.13 × rib eye area – 0.024 × carcass weight + 3.23.

^2^Yield grade: 30 = A; 20 = B; 10 = C.

^3^Marbling score: 1 = min; 9 = max.

^4^Quality grade: 50 = 1++; 40 = 1+; 30 = 1; 20 = 2; 10 = 3.

### Comparison of plasma BMP2 levels and expression of BMP2-adipogenesis signaling molecules in the LT between bulls and steers

**Figure 1:**
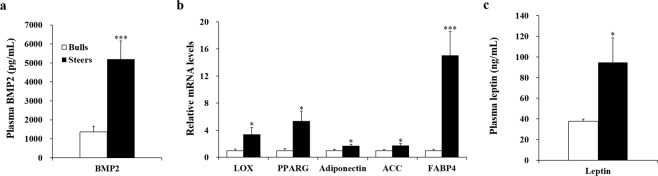
Plasma BMP2 and leptin levels and expression levels of LOX and adipogenic genes in Korean cattle bulls and steers. (**a,c**) The plasma BMP2 and leptin levels were measured using ELISA (n = 10/group). (**b**) mRNA levels in the *longissimus thoracis* were determined using quantitative PCR and normalized to β-actin (n = 10/group). mRNA levels in bulls were normalized to 1.0. Values are shown as the mean + standard error. *p < 0.05; ***p < 0.001. BMP2, bone morphogenetic protein 2; *LOX, lysyl oxidase; PPARG, peroxisome proliferator-activated receptor gamma; ACC, acetyl-CoA carboxylase; FABP4, fatty acid binding protein*.

Hyperplasia requires the proliferation and differentiation of preadipocytes into new adipocytes, a process known as adipogenesis^8,9,16^. BMPs are a group of growth factors in the transforming growth factor-beta superfamily^17^. BMP2 induces adipocyte development, including adipogenesis and preadipocyte commitment^10^. In this study, steers had higher (p < 0.001) plasma BMP2 concentrations than bulls (Fig. 1a). Consistent with our results, a study on humans found that circulating serum BMP2 levels were higher in a moderate obesity group than in lean healthy controls^18^. After castration of bulls, their circulating serum testosterone levels decreased^2^. A previously published mouse experiment showed that BMP signaling was blocked after testosterone injection^19^. Strong negative correlations have been observed between testosterone and body fat^20^. In this study, the decreased testosterone levels following castration may have triggered the higher circulating plasma BMP2 levels in steers, contributing to increased IMF deposition in castrated animals. Regression analysis revealed a positive association (p < 0.05) between plasma BMP2 levels and IMF content in the steer group, but not in the bull group (Table 2). The explanation for the absence of a significant association in the bull group may be that IMF content was relatively homogenous among animals in the bull group, as the standard deviation of IMF content in the bull group was much lower than that in the steer group (Table 1).

**Table 2:** Regression analysis of levels of plasma bone morphogenetic protein 2 (BMP2) and its signaling molecules in the *longissimus thoracis* with intramuscular fat content (IMF%) in Korean cattle.

Variables	Bull (n = 10)			Steer (n = 10)		
	Coefficient	*P* value	R^2^	Coefficient	*P* value	R^2^
Plasma leptin	−0.186	0.607	0.035	0.477	0.164	0.227
Plasma BMP2	−0.028	0.940	0.001	0.636	0.048	0.404
*LOX* mRNA level	0.209	0.563	0.044	−0.199	0.582	0.040
*PPARG* mRNA level	0.444	0.199	0.197	0.465	0.176	0.216
*Adiponectin* mRNA level	−0.160	0.659	0.026	−0.505	0.137	0.255
*ACC* mRNA level	0.593	0.071	0.352	−0.331	0.351	0.109
*FABP4* mRNA level	−0.120	0.741	0.014	−0.364	0.301	0.133

BMP2, bone morphogenetic protein 2; *LOX*, *lysyl oxidase; PPARG, peroxisome proliferator activated receptor gamma*; *ACC, acetyl-CoA carboxylase alpha; FABP4, fatty acid binding protein 4*.

The *LOX* gene is a downstream target of the BMP signaling pathway^10^. Steers had higher (p < 0.05) *LOX* mRNA levels in the LT than bulls (Fig. 1b). In mesenchymal stem cells derived from human adipose tissue, *LOX* expression was upregulated by BMP2 induction^21^. BMP2/4 induces the expression of *LOX*, which contributes to preadipocyte commitment by murine mesenchymal progenitor C3H10T1/2 cells^11^. Steers had higher (p < 0.05) *PPARG* mRNA levels in the LT than bulls. Steers also had higher (p < 0.05) *adiponectin* mRNA levels in the LT than bulls. In murine mesenchymal progenitor C3H10T1/2 cells, the induction of adipogenesis by BMP2 occurs through the activation of PPARG^22^. PPARG regulates the expression of several adipocyte-secreted proteins, including adiponectin^23^. In human mesenchymal cells (hMSCs), a combination of BMP2 and 3-isomethyl-1-methylxanthine induces adiponectin expression^24^. In our study, the up-regulation of BMP2 and *LOX* following castration may have led to the activation of preadipocyte commitment and subsequent adipocyte hyperplasia by adipogenesis through activation of PPAR and adiponectin in castrated animals. Steers had higher *acetyl CoA carboxylase* (p < 0.05) and *fatty acid binding protein 4* (p < 0.001) mRNA levels than bulls. Thus, de novo fatty acid synthesis and fatty acid transport may be subsequently activated through BMP signaling, resulting in increased IMF deposition in castrated animals.

### Comparison of expression levels of muscle fiber type and metabolism-related genes in the LT between bulls and steers

**Figure 2:**
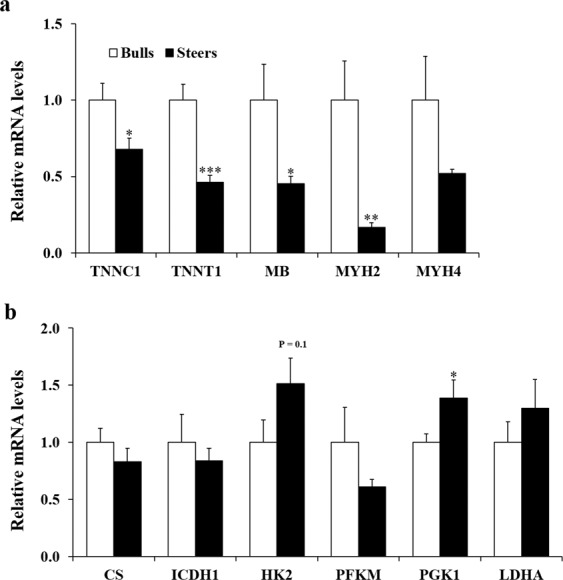
Expression levels of muscle fiber type and oxidative and glycolytic enzyme genes in the longissimus thoracis of Korean cattle bulls and steers. mRNA levels of muscle fiber type (**a**) and oxidative and glycolytic enzyme (**b**) genes were determined using quantitative PCR and normalized to β-actin (n = 10/group). mRNA levels in bulls were normalized to 1.0. Values are shown as the mean + standard error. *p < 0.05; **p < 0.01; ***p < 0.001. *TNNC1, troponin C1; TNNT1, troponin T1; MB, myoglobin; MYH2, myosin heavy chain 2; MYH4, myosin heavy chain 4; CS, citrate synthase; ICDH1, isocitrate dehydrogenase 1; HK2, hexokinase 2; PFKM, phosphofructokinase 1, muscle type; PGK1, phosphoglycerate kinase 1; LDHA, lactate dehydrogenase A*.

In our previous study, castration profoundly reduced circulating testosterone in cattle^2^. This endocrine change may affect muscle fiber type composition and muscle metabolism. In the present study, we checked whether castration affects the expression levels of genes related to muscle fiber type and muscle metabolism. Steers had lower mRNA levels of muscle fiber type 1 (slow twitch, red muscle, oxidative) genes, including *troponin C1* (p < 0.05), *troponin T1* (p < 0.001), and *myoglobin* (p < 0.05), than bulls (Fig. 2a). Previously, a lower proportion of type 1 fiber was observed in the *longissimus dorsi* muscle of steers compared to that of bulls in French Montbe´liard cattle at 16 months of age^15^. In our study, steers had lower (P < 0.01) mRNA levels of the fiber type 2A (fast twitch, oxidative, glycolytic) myosin heavy chain (MYH) isoform *MYH2* gene than bulls. mRNA levels of the fast type 2B (fast-twitch, glycolytic) *MYH4* gene did not differ (p > 0.05) between bulls and steers. Collectively, our results reveal that castration decreases transcription of muscle fiber types 1 and 2A genes in the LT.

These changes in fiber type gene expression may affect muscle metabolism. Thus, we compared the transcript levels of oxidative and glycolytic enzyme genes in the LT between bulls and steers. mRNA levels of oxidative enzyme genes, including *citrate synthase* and *isocitrate dehydrogenase 1*, did not differ between bulls and steers (Fig. 2b). Steers had higher (p < 0.05) mRNA levels of the glycolytic enzyme *phosphoglycerate kinase 1* gene than bulls (Fig. 2b). Steers also tended to have higher (p = 0.10) mRNA levels of the glycolytic enzyme *hexokinase 2* gene than bulls. mRNA levels of *muscle type phosphofructokinase 1* and *lactate dehydrogenase A* genes did not differ between bulls and steers. Consistent with our results, a previous study reported that steers exhibited higher glycolytic enzyme activities than bulls; however, the same study found that steers had lower oxidative enzyme activities than bulls, which contrasted with the results of the present study^15^. This inconsistency may be due to a difference in cattle breed (Korean cattle vs. French Montbe´liard cattle). In skeletal muscle forkhead box O1 transgenic mice, expression of muscle fiber type I genes, including slow isoforms of troponins and myoglobin, was down-regulated, but expression of mitochondrial oxidative enzyme genes involved in electron transport system (*cytochrome c oxidase II and IV*) did not differ, between forkhead box O1 mice and the control^25^. Collectively, our results indicate that castration moderately affects gene expression levels of glycolytic enzymes but does not affect gene expression of oxidative enzymes.

### Comparison of plasma leptin levels between bulls and steers

In this study, steers had higher (p < 0.05) plasma leptin concentrations than bulls (Fig. 1c). White adipose tissue is the main leptin-producing tissue^26^. Rodent primary culture experiments have shown that leptin is secreted after the differentiation of preadipocytes into adipocytes^27^. Consistent with our study, higher leptin concentrations were indicative of greater adiposity in beef cattle^28^. Therefore, higher body fat content in steers may be one explanation for the higher plasma leptin concentrations observed in our study and other studies. Positive correlations between adipocyte size and leptin expression have been found in human^29,30^ and rodent studies^31^. Similarly, plasma leptin concentrations are strongly related to adipose cell size in cattle^32^. Because larger adipocytes accommodate more leptin mRNA, adipocyte size may influence leptin synthesis and secretion^33^. Several studies have reported that hypertrophy is actively involved in marbling and IMF deposition^34,35^. Our previous study also revealed that castration induces hypertrophy of subcutaneous and abdominal fat in Korean cattle^36^. Therefore, we speculate that increased adipocyte hypertrophy may have contributed to the increased plasma leptin concentrations in castrated animals, although we did not measure the size of intramuscular adipocytes in this study.

## Conclusions

Our findings suggest that upregulation of the BMP signaling pathway in response to castration induces increased adipogenic gene expression in bulls, contributing to the increased IMF content observed in castrated animals. Our findings also indicate that castration affects the expression of some of muscle fiber type genes and has moderate effects on the expression of glycolytic pathway genes, but not oxidative enzymes. Our results reveal a new adipogenesis pathway for bovine IMF deposition.

## Materials and Methods

### Animals and tissue samples

All experimental procedures involving animals were approved by the Seoul National University Institutional Animal Care and Use Committee (SNUIACUC: SNU-161117-3) and conducted in accordance with the Animal Experimental Guidelines of the SNUIACUC. This study examined 10 Korean cattle bulls and 10 steers. We used bulls and steers, which have clear differences in IMF content^1^, as an experimental model to understand the involvement of BMP2 signaling molecules in IMF deposition and the metabolic differences between bulls and steers. A feeding regime was followed as previously described with modifications^2^. Briefly, 20 bulls were weaned at an average age of 3 mo and fed with 30% concentrates and 70% roughage until 6 mo of age. Ten bulls were castrated at 6 mo, the age at which Korean cattle bulls are routinely castrated. We castrated bulls under the guidance of an expert veterinarian. Bulls and steers were fed with concentrates composed of 15% crude protein (CP) and 71% total digestible nutrients (TDN) from 7 mo to 13 mo of age, 13% CP and 72% TDN until 19 mo of age, and 11% CP and 73% TDN after 20 mo of age. For roughage, timothy (2.5–3.5 kg/d; approximately 1.2% BW/animal) was fed from 7 mo to 13 mo of age, rice straw (2.0–3.0 kg/d; approximately 0.5% BW/animal) until 19 mo of age, and rice straw (1.0–1.5 kg/d; approximately 0.15% BW/animal) after 20 mo of age.

Before slaughter, blood was collected from the jugular vein into EDTA Vacutainer tubes, and plasma was prepared as previously described^37^. Bulls and steers were slaughtered at 25.7 ± 0.52 and 31.8 ± 0.11 months, respectively. Carcass traits, including MS, beef quality grade, ribeye area, fat thickness, yield index, and yield grade, were determined as previously described^2^. *Longissimus thoracis* (LT) muscles from the left carcass side between the 12^th^ and 13^th^ ribs were collected immediately after slaughter and stored at −70 °C until analysis. The IMF content of the LT muscles was measured following the procedure of Folch *et al*.^38^. Briefly, LT tissues were ground into a fine powder, which was homogenized in a 2:1 chloroform-methanol mixture (vol/vol). The fat content was measured after the fat-containing solvents were evaporated.

### Blood plasma analysis

The plasma BMP2 and leptin concentrations were quantified using enzyme-linked immunosorbent assays (ELISA) according to the manufacturer’s instructions. BMP2 was analyzed using a Bovine Bone Morphogenetic Protein 2 ELISA Kit (MyBioSource, San Diego, CA, USA) and leptin was analyzed using a Bovine Leptin ELISA Kit (MyBioSource). The intra- and inter-assay coefficients of variation for the Leptin and BMP2 kits were both less than 15%.

### RNA extraction and quantitative real-time polymerase chain reaction

Total RNA was extracted from LT tissue using TRIzol reagent (Molecular Research Center, Cincinnati, OH, USA), based on the manufacturer’s instructions. The RNA was quantified using a NanoPhotometer (Implen, Munich, Germany), and the quality was assessed using ethidium bromide staining of the 28S and 18S agarose gel electrophoresis bands and a Bioanalyzer (Agilent Technologies, Santa Clara, CA, USA), as previously described^39^. cDNA was synthesized from reverse-transcribed total RNA using an iScript cDNA Synthesis Kit (Bio-Rad Laboratories, Hercules, CA, USA), according to the manufacturer’s instructions.

Quantitative real-time PCR was performed using QuantiTect SYBR Green RT-PCR Master Mix (QIAGEN, Hilden, Germany), as previously described^39^. We followed the “Minimum Information for Publication of Quantitative Real-Time PCR Experiments” (MIQE) guidelines for qPCR as closely as possible^40^. All qPCR analyses were conducted in a 25-μL total reaction volume that contained 20 ng cDNA, 12.5 μL SYBR Green RT-PCR Master Mix, and 1.25 μL of 10 μM primers. The thermal cycling parameters were: 95 °C for 15 min, followed by 40 cycles at 94 °C for 15 s, 55 °C for 30 s, and 72 °C for 30 s. Table 3 lists the primers used. We used two different exons for forward and reverse primers to prevent amplification of the DNA template. We indicated the melting temperatures (Tm) of all primers. The Tms of all of the primers were 52.4–60.9 °C. An annealing temperature of 55 °C was used for amplification of all genes, resulting in a single major peak in all cases. The ΔΔCT method was used to determine the relative fold change in gene expression^41^. We evaluated whether β-actin, ribosomal protein lateral stalk subunit P0, and 18 s RNA were suitable reference genes. β-actin expression was generally uniform in the LT between bulls and steers and was therefore used as the reference gene. We also used β-actin as a reference gene in the LT in two previous studies^2,42^.

**Table 3:** Sequences of the primers used in real-time PCR analysis.

Gene name (Symbol)	Gene bank accession no.	Primer	Sequence (5′-3′)	Tm, °C	Length (bp)
β-actin (ACTB)^*^	NM_173979.3	Forward Reverse	AGCAAGCAGGAGTACGATGAGT ATCCAACCGACTGCTGTCA	60.5 58.0	120
*Lysyl oxidase (LOX)*	NM_173932.4	Forward Reverse	AACAATGTCGTCCGCTGTGA CCTTTGGGAGTTTTGGCTTGC	60.2 60.5	102
*Peroxisome proliferator activated receptor gamma (PPARG)*	NM_181024.2	Forward Reverse	AATCCCTGTTCCGTGCTGTG AAAGTTGGTGGGCCAAAACG	59.6 58.9	149
*Adiponectin*	NM_174742.2	Forward Reverse	CCCTGACTGAAGTCTGTGGC TCTTCCATGTTGTCCTCGCC	60.3 60.0	115
*Acetyl-CoA carboxylase alpha (ACC)*	NM_174224.2	Forward Reverse	AGACGTTGGAAGCAGAGAGG TTCAGCTCCAGAGGTTTGGC	59.4 55.0	142
*Fatty acid binding protein 4 (FABP4)*	NM_174314.2	Forward Reverse	GCTGCACTTCTTTCTCACCT TTCCTGGTAGCAAAGCCCAC	58.1 60.3	140
*Troponin C1, slow skeletal and cardiac type (TNNC1)*	NM_001034351.2	Forward Reverse	GTGAGCCGCCAGTATGGATG CACGAAGATGTCAAAGGCCG	60.9 59.6	97
*Troponin T1, slow skeletal type (TNNT1)*	XM_010815469.3	Forward Reverse	GCCTCTGAACATCGACCACA AGCTTCGCCATCAGGTCAAA	55.0 60.0	111
*Myoglobin (MB)*	NM_173881.2	Forward Reverse	GAGTCACATGCCAACAAGCAC GAAGTCTGAAGGATGCTTGGC	60.3 59.2	99
*Myosin heavy chain 2 (MYH2)*	NM_001166227.1	Forward Reverse	AAGAGCCCTTGGAATGAGGC ATGGCCATTTCCTGGTCCG	60.0 60.1	138
*Myosin, heavy chain 4, skeletal muscle (MYH4)*	NM_174224.2	Forward Reverse	GGTCCAAGTGCTGAAGAGGG GATGCAGCGTACAAAGTGGG	60.3 59.6	150
*Citrate synthase (CS)*	NM_001044721.1	Forward Reverse	CCATGGCTTTACTCACTGCG TTCGTGGAAGAAGCACTGGC	59.3 60.9	97
*Isocitrate dehydrogenase (NADP(+)) 1 (IDH1)*	NM_181012.3	Forward Reverse	TCCGAAATATCCTGGGTGGC CCCTGGCACAACAAAATCGG	59.5 60.0	149
*Hexokinase 2 (HK2)*	XM_015473383.2	Forward Reverse	TGCTCGCCTACTTCTTTACGG CCATCTCCTTGCGAAAACGC	60.1 60.2	126
*Phosphofructokinase, muscle (PFKM)*	NM_001075268.1	Forward Reverse	GGACAATCTGCAAAGAAGCCC CCACCAGAGGTTAACACGGC	52.4 60.0	126
*Phosphoglycerate kinase 1 (PGK1)*	NM_001034299.1	Forward Reverse	GTGGAGGAAGAAGGGAAGGG GAAAGTGAAGCTCGGAAGGC	59.4 59.2	89
*Lactate dehydrogenase A (LDHA)*	NM_174099.2	Forward Reverse	GCTATTAATCGGTGCCCCAGG TTGCCATCTTGGACTTAGACCC	60.9 60.0	110

^*^ACTB = Internal control gene.

### Statistical analyses

Differences between bulls and steers were examined using the general linear model procedure in SAS 9.1 software (SAS Institute, Cary, NC, USA). The IMF contents were not normally distributed due to marked differences between the bull and steer groups. Thus, we performed a linear regression analysis to separately examine the relationship between gene expression and IMF content (%) within the bull and steer groups using SAS 9.4 software. This resulted in the following equation:

$${\rm{IMF}} \% {\rm{i}}={\rm{\beta }}0+{\rm{\beta }}{\rm{1Expressioni}}+{\rm{\varepsilon }}{\rm{i}},$$

where IMF%i is the variable of IMF%, Expressioni is the variable expression level, β0 is the intercept, β1 is the coefficient of expression level, and εi is random error.
